# Berberine Ameliorates High-fat-induced Insulin Resistance in HepG2 Cells by Modulating PPARs Signaling Pathway

**DOI:** 10.2174/0115734099330183241008071642

**Published:** 2024-10-17

**Authors:** Lingxiao Zhang, Chenghao Yang, Xinyue Ding, Hui Zhang, Yuling Luan, Yueer Tang, Zongjun Liu

**Affiliations:** 1 Department of Cardiology, Putuo Hospital, Shanghai University of Traditional Chinese Medicine, Shanghai, China;; 2 Department of Cardiology, The Second People's Hospital of Fujian University of Traditional Chinese Medicine, Fuzhou, China;; 3 Institute of Cardiovascular Translational Medicine, Putuo Hospital, Shanghai University of Traditional Chinese Medicine, Shanghai, China

**Keywords:** Network pharmacology, insulin resistance, berberine, high-fat, peroxisome proliferator-activated receptor, ameliorates

## Abstract

**Background:**

Berberine (BBR), also known as berberine hydrochloride, was isolated from the rhizomes of the Coptis chinensis. Studies have reported that BBR plays an important role in glycolipid metabolism, including insulin resistance (IR). The targets, and molecular mechanisms of BBR against hyperlipid-induced IR is worthy to be further studied.

**Materials and Methods:**

The related targets of BBR were identified *via* Pharmmapper database and relevant targets of diabetes were obtained through GeneCards and Online Mendelian Inheritance in Man (OMIM) database. The common targets were employed with the STRING database and visualized with the protein-protein interactions (PPI) network. Gene Ontology (GO) and Kyoto Encyclopedia of Genes and Genomes (KEGG) enrichment analysis was performed to explore the biological progress and pathways. *In vitro*, human hepatocellular carcinomas (HepG2) cell was used as experimental cell line, and an insulin resistant HepG2 cell model (IR-HepG2) was constructed using free fatty acid induction. After intervention with BBR, glucose consumption and uptake in HepG2 cells were observed. Molecular docking was used to test the interaction between BBR and key targets, and real-time fluorescence quantitative PCR was used to detect the regulatory effect of BBR on related targets.

**Results:**

262 overlapped targets were extracted from BBR and diabetes. In the KEGG enrichment analysis, the peroxisome proliferator activated receptor (PPAR) signaling pathway was included. *In vitro* experiments, BBR can significantly increase sugar consumption and uptake in IR HepG2 cells, while PPAR inhibitors can weaken the effect of BBR on IR-HepG2.

**Conclusion:**

The PPAR signaling pathway is one of the important pathways for BBR to improve high-fat-induced insulin resistance in HepG2 cells.

## 1 INTRODUCTION

Insulin resistance (IR) is an important cause of the occurrence and development of type 2 diabetes [1], which runs through the whole process of disease development, and is a high risk factor for hypertension, obesity and cardiovascular diseases [2-4]. Researches have shown that the onset of insulin resistance often precedes the clinical diagnosis of type 2 diabetes [5], and the occurrence of diabetes-related complications has also increased. Insulin resistance often occurs in liver tissues, which not only seriously affects the production and utilization of glucose, but also affects the metabolism and synthesis of lipids [6]. Now the commonly used therapeutic drugs include metformin, insulin sensitizers, thiazolidinediones, *etc*. However, due to the potential risks of gastrointestinal reaction and adverse cardiac events, the development of safer and more reliable hypoglycemic drugs is still an important research field in the treatment of insulin resistance.

With the rapid development of traditional Chinese medicine, the effectiveness of traditional Chinese medicine in improving insulin resistance has been gradually confirmed, and the use of traditional Chinese medicine to improve insulin resistance to prevent and treat type 2 diabetes has become a hot research topic [7-10]. Berberine (BBR), an isoquinoline alkaloid extracted from Coptis chinensis and rhubarb [11, 12], has been used in traditional Chinese medicine to treat gastrointestinal inflammation for thousands of years.

Modern pharmacological studies have found that BBR has extensive pharmacological effects in the treatment of cardiovascular diseases, diabetes, tumor, inflammation, antibacterial and antiviral [13, 14]. A large number of clinical studies and animal experiments have shown that BBR has a role in alleviating insulin resistance [15], and its mechanism remains to be further studied.

Peroxisome proliferator activated receptor (PPAR), that is, peroxisome proliferator activated receptor, is divided into three subtypes, α, β/δ and γ, among which, PPARα is mainly distributed in tissues and organs such as liver, heart and skeletal muscle, which can promote energy dissipation and regulate glucose and lipid metabolism by affecting the transport, esterification and oxidation of fatty acids [16]. PPARγ can selectively promote lipid uptake and adipogenesis in adipose tissue, reduce the contents of circulating triglycerides and free fatty acids, and increase the insulin sensitivity of adipocytes [17].

Network pharmacology can integrate bioinformatics, pharmacology and biology to explore the complex mechanism of drug action on the human body in a “drug-disease-target-pathway” way [18]. Traditional Chinese herbs are characterized by multi-target and multi-pathway. In recent years, more and more studies have explored the mechanism of action of Chinese herbs through network pharmacology [19, 20]. The experimental verification of network pharmacology surpasses the limitation of single experimental study and can fully elucidate the mechanism of action of traditional Chinese medicine.

Therefore, in this study, combined with network pharmacological analysis and experimental verification, human hepatocellular carcinomas (HepG2) cells were induced by free fatty acid (FFA) to build an insulin resistance model, to elucidate the molecular mechanism of BBR regulating PPAR signaling pathway to improve insulin resistance, and providing a new therapeutic idea for the prevention and treatment of type 2 diabetes.

## 2 METHODOLOGY

### 2.1 Material

HepG2 cells were obtained from Shanghai Foresight Biological Co., LTD (BVCHUM113). Palmitic acid and BBR were purchased from Shanghai Aladdin Biochemical Technology Co.,LTD. PPARs inhibitor GW6471/GW9662 (HY-15372)/(HY-16578) were provided by Med ChemExpress. The PCR reaction kit was brought from Takara Baori Medical Biological Technology (Beijing) Co., Ltd. MEM Basic Medium was obtained from Thermo Fisher Biochemical Products Co., Ltd. Fetal bovine Serum was provided by Tianjin Kangyuan Biotechnology Co., Ltd. Penicillin-streptomycin dual antibody, PBS buffer solution, Corning Corporation, USA; Glucose test box was purchased from Nanjing Jiancheng Bioengineering Institute; CCK-8 kit, pancreatic enzyme cell digestive fluid and BCA protein concentration determination kit (enhanced) were offered from Shanghai Biyuntian Biotechnology Co., Ltd.

### 2.2 Network Pharmacology and Molecular Docking Technology 

Pharmmapper database (https://lilab-ecust.cn/pharmmapper/index.html) was used to predict potential targets of BBR. Diabetes related targets were obtained in Genecards database (https://www.genecards.org/) and OMIM database (https://www.omim.org/) and the target names were calibrated by the UniProt database (www.uniprot.org). Venny 2.1 (https://bioinfogp.cnb.csic.es/tools/venny/index.html) was used to analyze the targets obtained from the above database.

Protein-protein interaction (PPI) networks were mapped through the STRING database [21-23] (https://cn.string-db.org/). Metascape database (https://metascape.org/gp/index.html) was used to conduct online gene ontology (GO) enrichment analysis and Kyoto Encyclopedia of Genes and Genomes (KEGG) pathway enrichment analysis to study the biological functional processes and signaling pathways behind the core targets [24, 25].

The Autodock Tool was used for molecular docking verification [26], and the ligand molecules were drawn and converted into pdb format files by ChemDraw 20.0 [27]. The related structures of receptor molecules PPARγ (1KNU) and PPARα (2ZNN) were downloaded from the PDB database (https://www.rcsb.org/), and the target protein was dehydrated and hydrogenated by PyMol 2.4.1 to bind to the active site, and the binding energy was calculated [28]. It is generally believed that when the binding energy is less than 0 kcal/mol, the small ligand molecules can spontaneously bind to the receptor protein, and the lower the binding energy, the easier the binding and the more stable the conformation. When the binding energy is less than -5 kcal/mol, it is considered that there is a strong binding between the two.

### 2.3 HepG2 Cell Culture

HepG2 cells were frozen and resuscitated in a 37°C water bath. After centrifuge, cell precipitation was transferred to T25 culture bottle of 10% fetal bovine serum and 1% penicillin-streptomycin double resistant MEM base medium, and cultured in a constant temperature incubator at 37°C, 5% CO_2_, and 70%-80% humidity. After the cells were attached to the wall, the medium was sucked away, washed once with PBS, and appropriate amount of pancreatic enzyme was added for digestion for 2 min. After passage, the cells of logarithmic growth stage were taken for subsequent experiments at a ratio of 1:3.

### 2.4 Establishment and Grouping of IR-HepG2 Cell Models

The IR-HepG2 cell model was constructed based on the previous study [29]. After the intervention of HepG2 cells with 0.40 mM FFA for 24 hours, the cells were washed with 0.01 mol/L PBS solution pre-cooled at 4°C for 3 times to become a cell model with IR. The glucose content was detected with the glucose assay kit, and the total protein content was detected with the BCA protein concentration assay kit.

IR-HepG2 cell model was evaluated with glucose content. After successful model construction, BBR and GW6471/GW9662 were added, and the experimental groups were as follows: Normal Control group (Control group, MEM complete medium), Model group (Model group, 0.40 mM FFA +MEM complete medium), drug administration group (BBR group, 0.40 mM FFA+20 μM BBR+ MEM complete medium), Inhibitor group (Inhibitor group, 0.40 mM FFA+20 μM BBR+10 μM GW6471/GW9662 +MEM complete medium), positive control group (Metformin group, 0.40 mM FFA+20 μM metformin +MEM complete medium).

### 2.5 The Working Concentration of BBR was Screened by CCK-8

HepG2 cells were inoculated into 96-well plates at a density of 5000 cells/well, and the BBR solution was diluted to 0, 5, 10, 20, 40, 60, 80, 100 μM, 100 μL per well using MEM complete culture medium, with 6 multiple wells for each concentration, and cultured at 37°C and 5% CO_2_ for 24 hours. After the fresh medium was replaced, 10 μL CCK-8 solution was added to each well, and the absorbance was measured at 450 nm after continued incubation in the cell culture phase for 1 hour.

### 2.6 Glucose Consumption Test

HepG2 cells were inoculated into 96-well plates, with 5000 cells per well, and were divided into 5 groups according to 2.4. The cells were treated with medicine for 24 h after adhesion, and then 2.5 μL of culture medium supernatant from each well was applied for the test of glucose consumption. Strictly follow the instructions of the reagent kit to add samples to the 96-well plate, then gently shake the plate, incubate at 37°C for 10 minutes, and measure the absorbance value of each well at a wavelength of 505 nm using a microplate reader.

### 2.7 Glucose Uptake Assay

HepG2 cells were inoculated with 2.5×10^5^ cells/well in 24-well plates and grouped as described previously, and the drug administration group was given 20 μmol/LBBR. After 24 hours of treatment, the medium was discarded. After the cells were washed twice with PBS, 100 μmol/L 2-NBDG solution was added and incubated in a cell incubator at 37°C and 5% CO_2_ for 30min. After that, the cells were washed twice with pre-cooled PBS at 4°C. Images were taken and fluorescence intensity was quantitatively analyzed by ImageJ.

### 2.8 The mRNA Expressions of PPARA, PPRAG, GLUT1 and GLUT4 were Detected by Real-time Fluorescence Quantitative PCR

HepG2 cells at the logarithm growth stage were inoculated into 6-well plates with 1×10^6^ cells/well, and grouped as described previously. After 24 hours of culture, the TRlzol reagent was used to extract total RNA from the cells. RNA was reverse-transcribed into cDNA using PrimeScript™ RT reagent Kit with gDNA Eraser (Perfect Real Time) kit. Follow the instructions for the TB Green^®^ Premix Ex Taq™ kit to perform the qRT-PCR reaction. Reaction conditions: predenaturation at 95°C for 30 s; Denaturation at 95°C for 5 s, annealing/extension at 60°C for 20 s, a total of 40 cycles. The relative mRNA expression of related genes was calculated by the 2^-∆ ∆Ct^ method. RT-PCR Amplified primer sequences are shown in Table **1**.

### 2.9 Statistic Analysis of Data

All experiments were repeated at least three times, and the representative results are presented. Statistics and plotting were performed using GraphPad Prism 8.0.10. Data were expressed in the form of mean ± standard deviation. One-Way ANOVA was used among multiple groups, and LSD test was used for comparison between the two groups. It was generally believed that *p* <0.05 meant that the difference was statistically significant.

## 3 RESULTS

### 3.1 Anti-diabetic Target Prediction of BBR based on Network Pharmacology and Molecular Docking Techniques

**Fig. (1):**
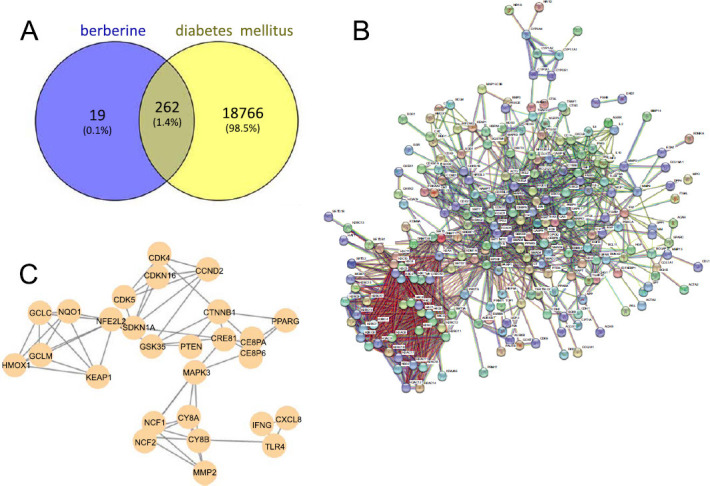
The common targets of BBR and diabetes were screened, and the GO and KEGG pathways were enriched for the target genes. (**A**) A common target for BBR and diabetes was identified. (**B** and **C**) Protein-protein interaction (PPI) network of shared targets.

A total of 281 targets for BBR were obtained from PharmMapper and a total of 19,028 potential targets for diabetes were collected from databases such as GeneCards. Finally, 262 targets were identified as common targets for BBR and diabetes (Fig. **1A**), these targets were constructed with PPI networks (Fig. **1B** and **C**).

**Fig. (2):**
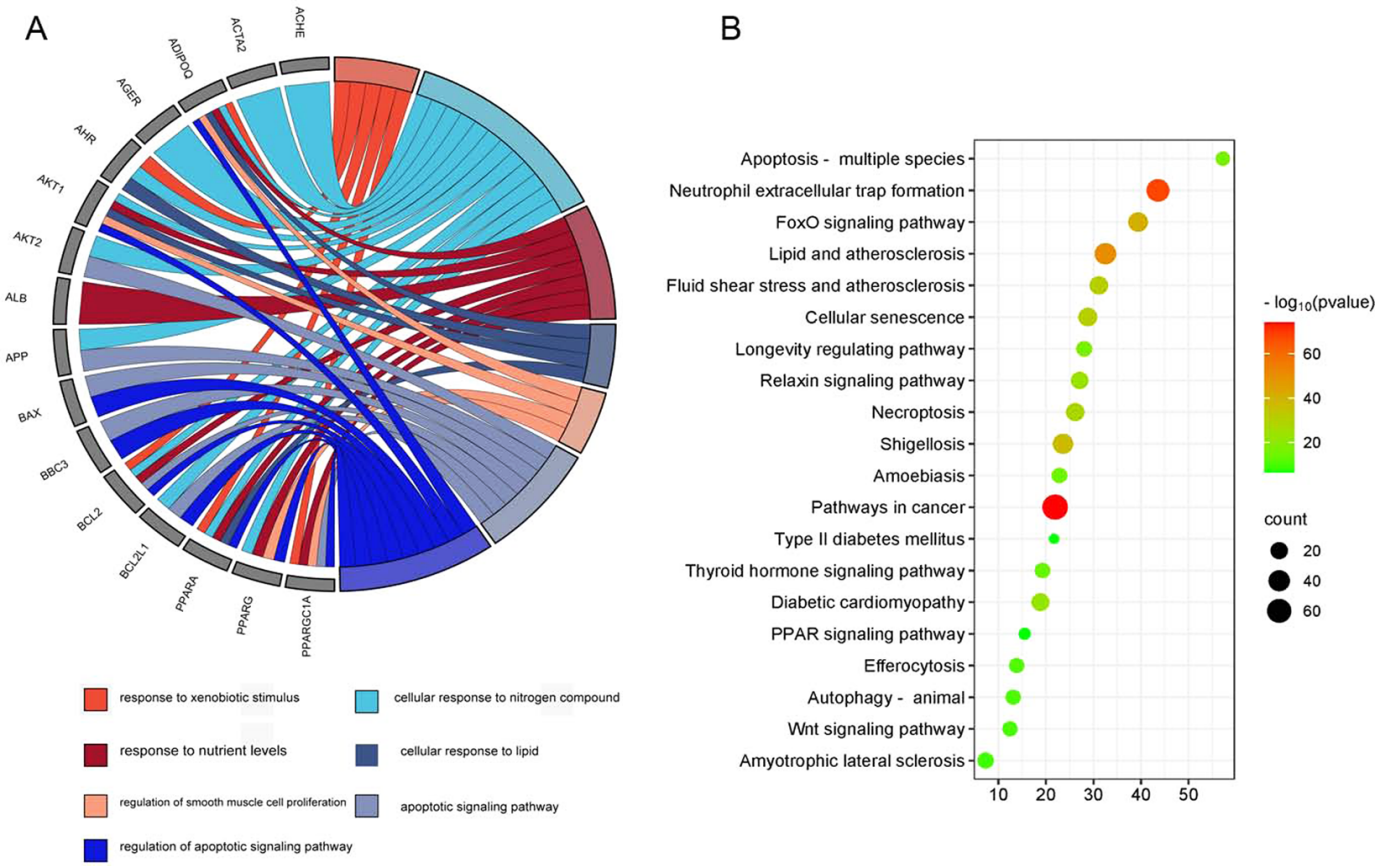
GO and KEGG analysis of target genes. (**A**) GO analysis of bioenrichment processes (BP) at common targets was performed. (**B**) KEGG analysis of common target enrichment signaling pathways.

GO enrichment analysis showed that common targets were mainly related to terms such as “cell response to lipids” and “response to foreign stimuli” (Fig. **2A**). The results of KEGG pathway enrichment analysis showed that potential targets were related to “lipid and atherosclerosis”, “FoxO signaling pathway”, “diabetic cardiomyopathy”, “PPAR signaling pathway”, *etc*. (Fig. **2B**).

The ability of a protein to bind to a potential compound with high affinity is key to finding a target. To further understand the effect of BBR on insulin resistance in HepG2 cells through the regulation of PPARs, molecular docking was performed by Autodock Tool in this study, and the results are shown in Fig. (**3**). The binding energy of BBR to PPARγ is -10.26 kcal/mol, while the binding energy of PPARα is -9.19 kcal/mol. BBR can be associated with LEU-255, MET-348, LYS-263, and other amino acid residues in PPARγ, among which GLU-291 and HIS-266 can be hydrogen bonded in three ways. BBR can bind to ASN-219, GLU-282, VAL-324 and other amino acid residues in PPARα, and three hydrogen bonds can be formed with CYS-278, GLY-335, and LEU-331. Therefore, BBR is a potent bioactive compound that can act on the PPARs signaling pathway.

### 3.2 BBR Working Concentration Screening

The effects of berberine at different concentrations on HepG2 cell viability were detected by CCK-8, as shown in Fig. (**4A**). When BBR concentration does not exceed 40μMol/L, BBR has no significant inhibitory effect on HepG2 cell viability, and when BBR concentration exceeds 40μMol/L, HepG2 cell viability decreases with the increase of BBR concentration. Therefore, according to the determination of CCK-8 experiment results, we used 20μMol/L BBR as the working concentration for follow-up experiments.

### 3.3 Effect of BBR on Glucose Consumption in HepG2 Cells

Results As shown in Fig. (**4B**), compared with the control group, glucose consumption of HepG2 cells in the model group decreased significantly (*p* <0.001), indicating successful cell modeling. Compared with the model group, the glucose consumption of HepG2 cells increased after BBR administration (*p* <0.001). With metformin as a positive control drug, there was no significant difference in glucose consumption of HepG2 cells between the BBR group and the metformin group (*p* >0.05). Compared with the BBR group, the glucose consumption of HepG2 cells in the inhibitor group was significantly decreased (*p* <0.05).

The effect of BBR on glucose uptake in IR-HepG2 cells was observed by an inverted fluorescence microscope. Relatively clear green fluorescence can be observed under a 10× field of view, and the fluorescence intensity quantified by ImageJ is compared as shown in Fig. (**5**). Compared with the control group, the fluorescence intensity of the model group was weaker (*p* <0.01), while the fluorescence intensity of the BBR group was stronger than that of the model group (*p* <0.05), and the fluorescence intensity of the metformin group was not significantly different from that of the BBR group (*p* >0.05). The fluorescence intensity of inhibitor group was significantly decreased compared with BBR group (*p* <0.05).

### 3.4 Effect of BBR on mRNA Expression in IR-HepG2 Cells

Validation was performed at the transcriptome level by RT-PCR. As shown in Fig. (**6**), PPAR mRNA was significantly decreased in the model group (*p* <0.01), while the BBR group up-regulated PPARα, PPARγ and GLUT4 mRNA levels in IR-HepG2 cells. The effect of BBR on GlUT4 in IR-HepG2 cells was attenuated by the administration of inhibitors GW6471/GW9662. GLUT1 was significantly increased in the model group, BBR could significantly down-regulate the expression of GLUT1 mRNA (*p* <0.01), and the effect of BBR on GLUT1 mRNA was weakened after the addition of inhibitor (*p* <0.05). Conclusion: BBR can improve FFA-induced insulin resistance by regulating PPARα, PPARγ, and GLUT4 mRNA levels as described above.

## 4 DISCUSSION

With the change of people's lifestyle, such as the reduction of exercise and the increase of high-fat diet, the incidence of diabetes is increasing year by year. It has been found that obesity increases the risk of type 2 diabetes mellitus (T2DM) [30], and insulin resistance caused by obesity in pre-type 2 diabetes is one of the main causes of T2DM [31]. Therefore, it is an urgent problem to seek effective prevention and treatment of insulin resistance induced by high fat.

As a safe and effective traditional medical means, Chinese medicine has a good synergistic effect in the prevention and treatment of diabetes [32, 33]. BBR is the most representative active ingredient in Coptis, and has great potential in the treatment of diabetes [34]. Studies have shown that BBR can reduce the level of inflammatory factors to reduce the apoptosis of islet B cells [35]. Current research has reported that BBR activates AMPK phosphorylation in 3T3-L1 adipocytes, thereby enhancing the glucose transport capacity of glucose transporter 1 (GLUT1) and increasing the uptake of glucose by tissues [36]. BBR inhibits sarcopenia-induced insulin resistance through SIRT1-mediated mitochondrial autophagy [37]. BBR can also improve the level of oxidative stress and maintain the stability of blood sugar by activating MAPK signaling pathway [38]. Moreover, it can regulate intestinal flora, reduce endotoxin levels, and play a role in lowering glucose and regulating lipid [39]. Most importantly, BBR can also increase insulin sensitivity and improve insulin resistance [40, 41].

There are many mechanisms through which Chinese medicine can prevent and cure diabetes, and it is one of the classic angles to study the prevention and cure of insulin resistance by Chinese medicine through PPARs related pathway [42-44]. Previous study have shown that BBR increased the expression of PPARα and carnitine palmitoyl transferase 1 (cpt-1) genes in Oreochromis niloticus feeding with a high-fat diet, leading to a decrease in lipid accumulation [45]. Therefore, this research studied the mechanism of BBR improving insulin resistance based on PPARs signaling pathway. Using network pharmacology, we initially predicted the potential molecular mechanisms regulated by BBR, particularly its impact on the PPAR signaling pathway. Subsequently, through molecular docking experiments, we further confirmed that BBR exhibits excellent binding affinities with both PPARα and PPARγ, providing a solid structural foundation for BBR to function through this signaling pathway. To directly validate this hypothesis, we established an I/R-HepG2 cell model, which mimics the state of insulin resistance (IR). In this model, we observed that BBR significantly enhanced the cells' glucose uptake and absorption capabilities. To elucidate the pivotal role of the PPAR signaling pathway in BBR's amelioration of insulin resistance, we introduced PPAR-specific inhibitors. The experimental results revealed that when the PPAR signaling pathway was blocked, the beneficial effects of BBR on insulin resistance were markedly diminished. This crucial finding directly supports our hypothesis that BBR effectively addresses FFA -induced insulin resistance by activating the PPAR signaling pathway.

In summary, our study not only unveils the close association between BBR and the PPAR signaling pathway at the molecular level but also validates, through cellular experiments, the specific mechanism of BBR's improvement of insulin resistance *via* this pathway. This achievement not only deepens our understanding of BBR's pharmacological effects but also provides a robust scientific basis and potential target directions for the development of novel diabetic therapies.

Furthermore, this result should be better validated in *in vivo* models, and more data is needed to elucidate how BBR modulates the PPARs signaling pathway during insulin resistance (I/R), which was not thoroughly explored in this study. This will be one of our future research directions.

## CONCLUSION

In conclusion, a total of 262 overlapped targets were extracted from BBR and diabetes. PPAR signaling pathways were found to be potentially primarily responsible for BBR against hyperlipid-induced IR. Vitro experiments provides a view of the potential pharmacological mechanisms for BBR can significantly increase sugar consumption and uptake in IR HepG2 cells. The study indicates that BBR may serve as a promising complementary and alternative drug for diabetes but needs further *in vivo* experiments.

## AUTHORS’ CONTRIBUTIONS

Study conception and design: C.Y; draft manuscript and perform experiment: L.Z and X.D; methodology: H.Z; data analysis: Y.L and Y.T; Z.L supervised the study; All authors reviewed the results and approved the final version of the manuscript.

## Figures and Tables

**Fig. (3):**
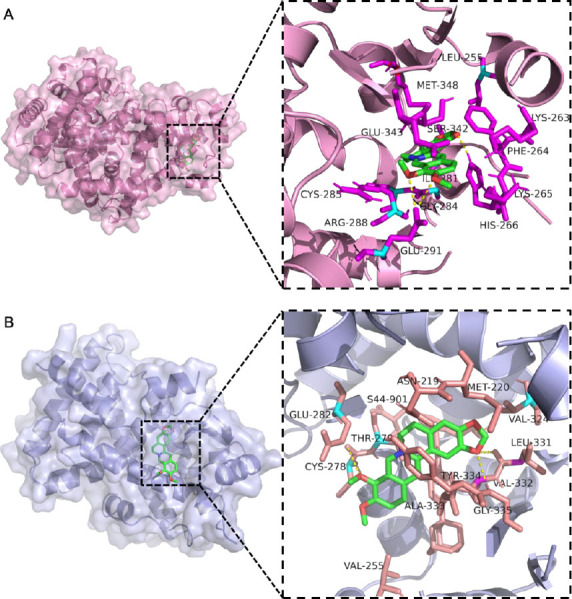
Docking results of berberine with PPARs molecules. (**A**) Docking result of berberine with PPARγ. (**B**) Docking result of berberine with PPARα.

**Fig. (4):**
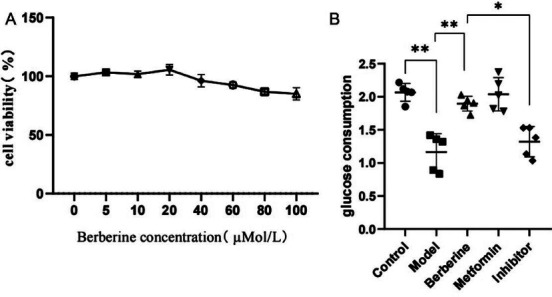
Effects of BBR on the viability and glucose consumption of HEPG2 cells. (**A**)The effect of different concentrations of berberine on the cell viability of HEPG2. n=6. (**B**) Effect of berberine on glucose consumption of HepG2. *p <0.05, **p < 0.01, n=5.

**Fig. (5):**
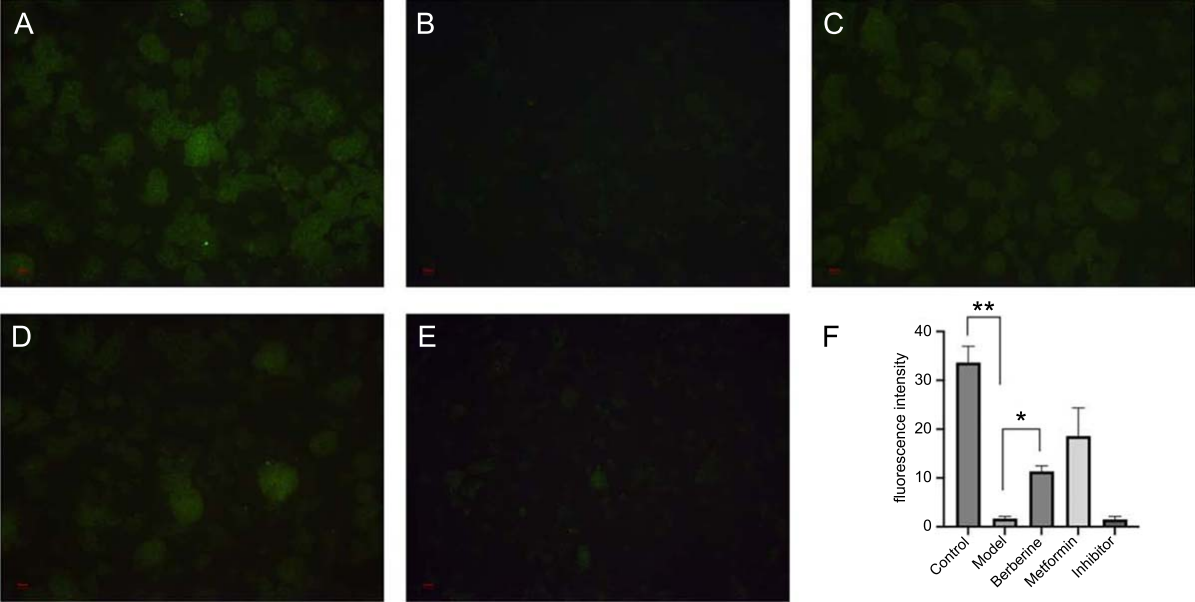
The effect of BBR on glucose uptake in HepG2 cells. (**A**) Control group, (**B**) Model group, (**C**) Metformin group, (**D**) BBR group, (**E**) Inhibitor group, (**F**) the analysis of fluorescence intensity. *p <0.05, **p < 0.01, n=5.

**Fig. (6):**
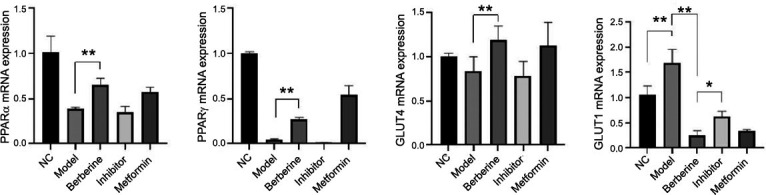
The effect of BBR on the expression of mRNA related to IR-HepG2 cells. *p <0.05, **p < 0.01, n=5.

**Table 1:** RT-PCR Amplified primer sequence.

**Gene symbol**	**-**	**Sequence (5' -> 3')**
PPARA	Forword	CGGTGACTTATCCTGTGGTCC
	Reverse	CCGCAGATTCTACATTCGATGTT
PPARG	Forword	TACTGTCGGTTTCAGAAATGCC
	Reverse	GTCAGCGGACTCTGGATTCAG
GLUT1	Forword	CTGTGCTCCTGGTTCTG
	Reverse	CTGTGCTCCTGGTTCTG
GLUT4	Forword	TTGGAAGGAAAAGGG
	Reverse	GCAGGTGAGTGGGAG
GAPDH	Forword	GGAGCGAGATCCCTCCAAAAT
	Reverse	GGCTGTTGTCATACTTCTCATGG

## Data Availability

The data and supportive information is available within the article.

## LIST OF ABBREVIATIONS

BBR: Berberine
FFA: Free Fatty Acid
GLUT1: Glucose Transporter 1
GLUT4: Glucose Transporter 4
GO: Gene Ontology
HepG2: Human Hepatocellular Carcinomas
IR: Insulin Resistance
IR-HepG2: Insulin Resistant HepG2 Cell Model
KEGG: Kyoto Encyclopedia of Genes and Genomes
OMIM: Online Mendelian Inheritance in Man
PPAR: Peroxisome Proliferator Activated Receptor
T2DM: Type 2 Diabetes Mellitus
